# The Gender Impact on Morphogenetic Variability in Coronary Artery Disease: A Preliminary Study

**DOI:** 10.3390/jcm7050103

**Published:** 2018-05-03

**Authors:** Radmila Karan, Biljana Obrenovic-Kircanski, Suzana Cvjeticanin, Natasa Kovacevic-Kostic, Milos Velinovic, Vladimir Milicevic, Milica Vranes-Stoimirov, Dejan Nikolic

**Affiliations:** 1 Faculty of Medicine, University of Belgrade, Koste Todorovic 8, Belgrade 11000, Serbia; biljanaok@yahoo.com (B.O.-K.); cujasimsi@gmail.com (S.C.); cipelcici@yahoo.com (N.K.-K.); velinovicsurg@gmail.com (M.V.); denikol27@gmail.com (D.N.); 2 Department of Anesthesiology and Intensive Care, Clinic for Cardiac Surgery, Clinical Center of Serbia, Belgrade 11000, Serbia; 3 Clinic for Cardiology, Clinical Center of Serbia, Belgrade 11000, Serbia; 4 Institute for Human Genetics, Faculty of Medicine, University of Belgrade, Belgrade 11000, Serbia; 5 Clinic for Cardiac Surgery, Clinical Center of Serbia, Belgrade 11000, Serbia; vladodrillwork@gmail.com; 6 Clinic for Vascular and Endovascular Surgery, Clinical Center of Serbia, Belgrade 11000, Serbia; rkaran@sbb.rs; 7 Department of Physical Medicine and Rehabilitation, University Children’s Hospital, Belgrade 11000, Serbia

**Keywords:** coronary artery disease, homozygous recessive characteristics, variability, gender

## Abstract

We analyzed morphogenetic variability and degree of genetic homozygosity in male and female individuals with coronary artery disease (CAD) versus unaffected controls. We have tested 235 CAD patients; 109 were diagnosed also with diabetes mellitus (DM) and 126 with hypertension (HTN). We additionally evaluated 152 healthy individuals without manifested CAD. For the evaluation of the degree of recessive homozygosity, we have performed the homozygously recessive characteristics (HRC) test and tested 19 HRCs. In controls, the frequency of HRC for males was 2.88 ± 1.89, while for females, it was 3.65 ± 1.60. In the CAD group, the frequency of HRC for males was 4.21 ± 1.47, while for females, it was 4.73 ± 1.60. There is significant difference in HRC frequencies between controls and CAD separately for males (*p* < 0.001) and females (*p* < 0.001). The same applies between controls and CAD with DM (males: *p* < 0.001 and females: *p* = 0.004), and controls and CAD with HTN (males: *p* < 0.001 and females: *p* < 0.001). There is no significant difference in HRC frequencies between the group of CAD with DM and the group of CAD with HTN (males: *p* = 0.952 and females: *p* = 0.529). Our findings point to the increased degree of recessive homozygosity and decreased variability in both genders of CAD patients versus controls, indicating the potential genetic predisposition for CAD.

## 1. Introduction

Despite the fact that cardiovascular disease (CVD) was long thought to be a disease of the male gender, recent studies suggest that it is more common in women with certain risk factors (RF) that are unique to the female gender (gestational diabetes and hypertension) [1]. Due to the fact that the female gender is less homogenous in terms of hormonal effects on CVD, particularly in their reproductive years, so far, it has been delicate to perform a gender-balanced study for CVD [2]. Such claims are justified by the difference in physiology and pharmacokinetics, and pharmacodynamics in females versus males. The recent study by Reckelhoff stressed that hypertension awareness is greater in women, while hypertension itself is more common in men than in women in the reproductive period. Also, an important point is that there are different mechanisms responsible for blood pressure control between the male and female genders [3]. In previous studies on population genetic level, the difference between genders with spina bifida was established, contributing to better understanding of etiopathogenetic mechanisms [4,5].

Given the facts above, the presence of gender differences and possible genetic predisposition led us to hypothesize that increased genetic homozygosity and decreased variability in tested individuals of different genders with coronary artery disease (CAD) could be morphogenetic parameters for the prediction of such a state. Therefore, we aimed to analyze and compare morphogenetic variability and the degree of genetic homozygosity in male and female individuals with CAD versus unaffected controls.

## 2. Material and Methods

### 2.1. Study Group

We have tested 235 CAD patients between 2015 and 2017, of which 109 were also diagnosed with diabetes mellitus (DM), and 126 that were also diagnosed with hypertension (HTN). 

For comparison, we additionally evaluated 152 healthy individuals without manifested CAD. The tested individuals with CAD and the control group belong to the same population (Serbian population) of similar socioeconomic status and age (between 56–65 years). The study was conducted according to the principles of good clinical practice and followed the recommendations of the declaration of Helsinki. The study was approved by relevant Institutional Review Board. According to gender, all tested individuals were divided into two groups: male and female.

Prior to inclusion in the study, patients were informed about study protocol and informed consent was obtained.

Hypertension was defined as a systolic blood pressure (SBP) ≥ 140 mm Hg or a diastolic blood pressure (DBP) ≥ 90 mm Hg [6]. Diabetes mellitus was defined as a fasting plasma glucose level (FPG) ≥ 7.0 mmol/L or random plasma glucose level ≥ 11.1 mmol/L [7].

### 2.2. Tested Determinants

For the evaluation of the degree of recessive homozygosity, we have performed the homozygously recessive characteristics (HRC) test, which is used to establish the proportion of homozygously recessive clearly expressed characteristics in every individual as markers of chromosomal homozygosities, implicating the degree of genetic homozygosity in humans [8,9,10]. HRC testing is based on an analysis of numerous morphophysiological traits with a known genetic determination. These evaluated homozygous traits are controlled by genes found on different chromosomes and can be treated as markers of the same chromosomes, including surrounding genes that control different elements of fitness. The degree of homozygosity established by the HRC test basically represents an estimate of genetic loads present in a certain group of the human population being tested or the human population in general [11,12,13].

We have evaluated 19 HRCs in every studied individual, where only characteristics with extreme appearance were marked as a present trait: attached ear lobe (OMIM number 128900), continuous frontal hair line (OMIM number 194000), blue eyes (gene location 15q12, 15q13, OMIM number 227220; 5p13 OMIM number 227240; 14q32.1, OMIM number 210750; 9q23 OMIM number 612271), straight hair (1q21.3, OMIM number 139450), soft hair and blond hair (gene location 15q12, 15q13, OMIM number 227220; 14q32.1, OMIM number 210750; 12q21.3 OMIM number 611664; 11q13.3, OMIM number 612267), double hair whorl, opposite hair whorl orientation (OMIM number 139400), as well as an inability to roll, fold, and curve the tongue (OMIM number 189300), ear without Darwinian notch, and a guttural “r” in speech, proximal thumb hyperextensibility, index finger longer than the ring finger (OMIM number 136100), left-handedness (gene location 2p12-q22, OMIM number 139900), right thumb over left thumb (hand clasping) (OMIM number 139800), top joint of the thumb > 45°, and three tendons in the wrist (OMIM) [14].

### 2.3. Statistical Analysis

The results were presented as whole numbers and percentages, while continuous variables were presented as mean value ± standard deviation and 95% confidence interval (CI). For comparison among groups, we performed the Mann–Whitney U Test. Variation coefficient (V) was used to compare variability between the evaluated groups of individuals. We used SPSS version 17.0 for statistical evaluation. Statistical significance was set as *p* < 0.05.

## 3. Results

From 235 patients with CAD, 109 (46.38%) had DM and 126 (53.62%) had HTN. There were 111 (47.23%) male and 124 (52.77%) female patients with CAD. From those with DM, 52 (47.71%) were males and 57 (52.29%) females; while from those with HTN, 59 (46.83%) were males and 67 (53.17%) were females.

All evaluated patients were from a Serbian population between 56–65 years of age. From 152 individuals in control group, there were 77 (50.66%) males and 75 (49.34%) females. The control group consisted of individuals from same locality (Serbian population), of similar age (56–65 years), and with similar socioeconomic status as patients with CAD.

**Figure 1:**
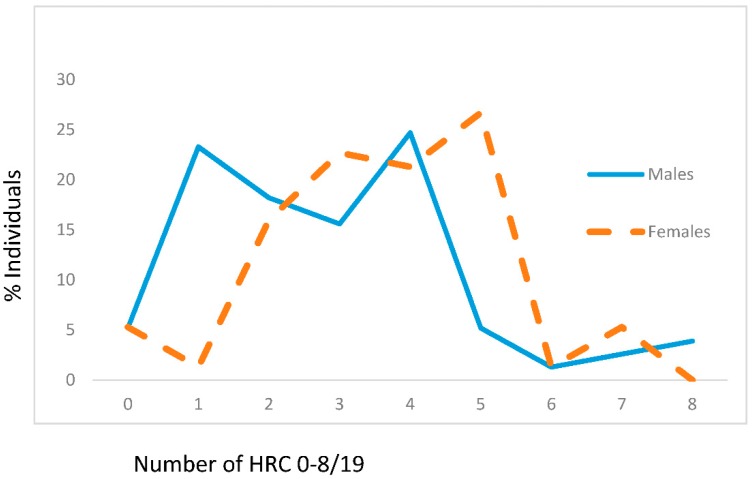
Frequencies of homozygous recessive characteristics (hrc) in males and females of the control group. X—mean values with standard deviation, V—variability, z—Mann Whitney *U* test. Males: *n* = 77, x_hrc/19_ = 2.88 ± 1.89 (95% CI 2.42–3.28). Females: *n* = 75, x_hrc/19_ = 3.65 ± 1.60 (95% CI 3.29–4.02; z = −3.164, *p* = 0.002). V_Males_ = 65.63%, V_Females_ = 43.84%.

Presented findings showed that mean values of HRC significantly differed between male and female genders in the control group (male gender: 2.88 ± 1.89, female gender: 3.65 ± 1.60, z = −3.164, *p* = 0.002) (Figure 1). In the control group of individuals, the most frequent average number of HRC was 4 (around 25%) for males and 5 (slightly above 25%) for females (Figure 1).

**Figure 2:**
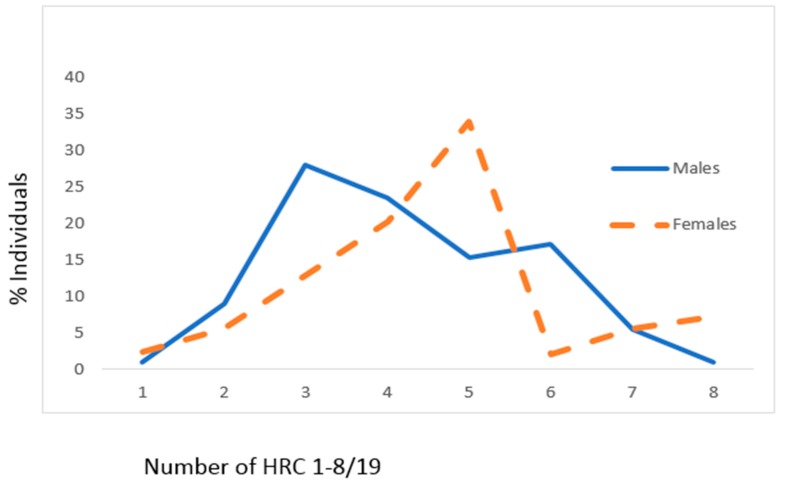
Frequencies of homozygous recessive characteristics (hrc) in males and females of the CAD sample. X—mean values with standard deviation, V—variability, z—Mann Whitney *U* test. Males: *n* = 111, x_hrc/19_ = 4.21 ± 1.47 (95% CI 3.93–4.48). Females: *n* = 124, x_hrc/19_ = 4.73 ± 1.60 (95% CI 4.44–5.00; z = 2.564, *p* = 0.010). V_Males_ = 34.92%, V_Females_ = 33.83%.

For the group of CAD patients, mean values of HRC significantly differed between male and female genders (male gender: 4.21 ± 1.47, female gender: 4.73 ± 1.60, z = 2.564, *p* = 0.010) (Figure 2). In the CAD group of individuals, the most frequent average number of HRC was 3 (just above quarter) for males and 5 (slightly above 1/3) for females (Figure 2).

**Figure 3:**
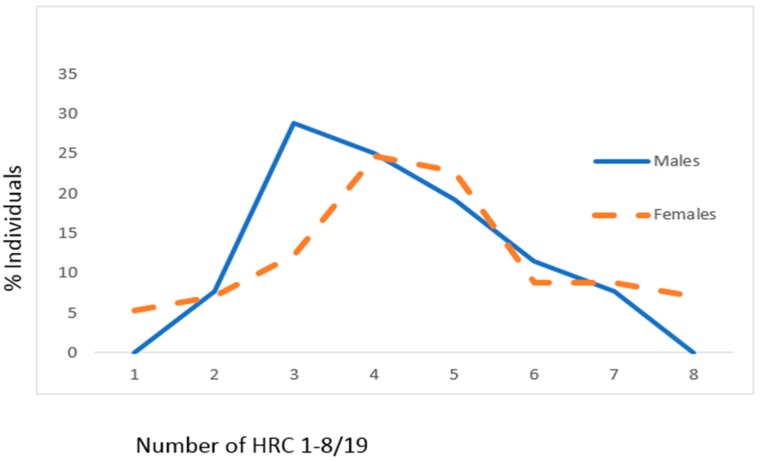
Frequencies of homozygous recessive characteristics (hrc) in males and females of the CAD sample with DM. X—mean values with standard deviation, V—variability, z—Mann Whitney *U* test. Males: *n* = 52, x_hrc/19_ = 4.21 ± 1.39 (95% CI 3.82–4.60). Females: *n* = 57, x_hrc/19_ = 4.67 ± 1.88 (95% CI 4.06–5.03; z = 1.401, *p* = 0.162). V_Males_ = 33.02%, V_Females_ = 40.26%.

In CAD patients with DM, mean values of HRC did not significantly differ between male and female genders (male gender: 4.21 ± 1.39, female gender: 4.67 ± 1.88, z = 1.401, *p* = 0.162) (Figure 3). In CAD patients with DM, the most frequent average number of HRC was 3 (around 30%) for males and 4 (slightly below 25%) for females (Figure 3).

**Figure 4:**
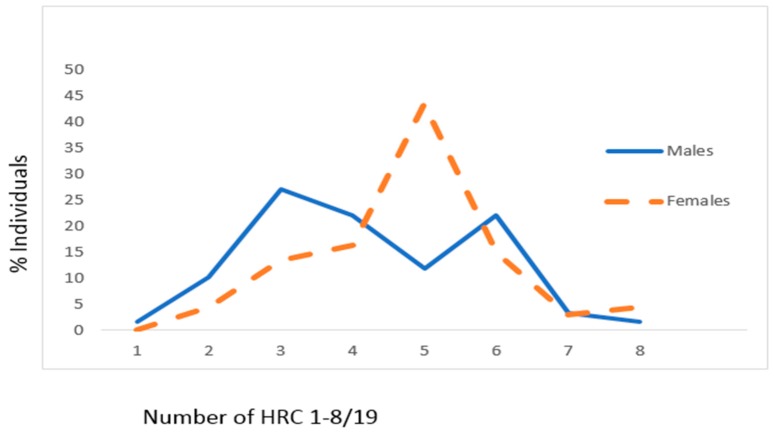
Frequencies of homozygous recessive characteristics (hrc) in males and females of the CAD sample with HTN. X—mean values with standard deviation, V—variability, z—Mann Whitney *U* test. Males: *n* = 59, x_hrc/19_ = 4.20 ± 1.55 (95% CI 3.80–4.61). Females: *n* = 67, x_hrc/19_ = 4.78 ± 1.32 (95% CI 4.45–5.10; z = 2.075, *p* = 0.038). V_Males_ = 36.90%, V_Females_ = 27.62%.

Presented findings showed that mean values of HRC significantly differed between male and female genders in CAD patients with HTN (male gender: 4.20 ± 1.55, female gender: 4.78 ± 1.32, z = 2.074, *p* = 0.038) (Figure 4). In CAD patients with HTN, the most frequent average number of HRC was 3 (around 27%) for males and 5 (slightly above 40%) for females (Figure 4).

There is a significant difference in HRC frequencies between the controls and CAD group separately for males (*p* < 0.001) and females (*p* < 0.001) (Table 1). The same applies between the controls and CAD group with DM (males: *p* < 0.001 and females: *p* = 0.004), and the controls and CAD group with HTN (males: *p* < 0.001 and females: *p* < 0.001) (Table 1). There is no significant difference in HRC frequencies between the group of CAD with DM and the group of CAD with HTN (males: *p* = 0.952 and females: *p* = 0.529) (Table 1).

## 4. Discussion

Estimation of genetic homozygosity in the human population is a delicate task, due to the fact that only a small number of loci with allelic genes are presently identified [15]. It is assumed as well that given the type of inheritance and variability, HRCs might be controlled by one or several genes that are located on various human chromosomes [15].

Results of our study demonstrated the presence of a significant difference in the degree of recessive homozygosity between genders for both the control group and the CAD group. However, it is obvious that there is a presence of a significantly increased degree of recessive homozygosity between males in the CAD group versus the control group, as well as females in the CAD group versus the control group. In the control group, in males, most frequently 4 HRCs were present (one out of four), while in females, it was 5 HRCs (one out of four). In the CAD group, in males, most frequently 3 HRCs were present (one out of four), whereas females had 5 HRCs most frequently (one out of three). Another point to stress is the decreased variability in males and females in the CAD group versus males and females in the control group, with the decrease being greater in males than females. Such a finding might point to an assumption that there could be a population genetics difference between genders for the predisposition of CAD. These findings could argue, to a certain degree, for the hypothesis that increased homozygosity may lead to decreased body resistance. This could justify the premise that genes determining evaluated recessive traits together with environmental factors might potentially influence the development of a certain condition, particularly in our study for CAD, more in females [16]. Such findings are in line with previous reports, which have stressed that females have specific factors unique to the gender along with gender-specific mechanisms responsible for blood pressure control [1,3]. Along with these claims, it should be stressed as well that increased genetic homozygosity in the CAD sample of patients, in particular in the female gender, might have an effect on genes that is assumed to be responsible for the expression of such a state, thus increasing susceptibility. Decreased variability corresponds with narrower variations of genetic homeostasis, meaning that certain extreme genotypes have a higher tendency to appear in a human individual, thus exposing that individual to a greater risk of developing CAD.

The significant importance of DM on CAD has been stressed previously, noticing that CAD is considered an important factor of long-term prognosis in patients that were diagnosed with DM, where these individuals have increased mortality risk from heart disease [17]. Furthermore, the genetic background of DM was stressed in previous studies, implicating that it is heterogeneous, so type 2 DM could be divided into monogenic and polygenic forms [18,19,20]. So far, numerous genes have been reported to be associated with DM susceptibility (11), among them also genes on the X chromosome: Xp11 (OMIM number 300136), Xq28 (OMIM number 304800), and Xp11.23 (OMIM number 304790) [14]. Our findings demonstrated increased genetic homozygosity in females versus males, but not significantly in the CAD group with DM, whereas variability in females increased versus males. Despite the fact that we found increased homozygosity in females, the absence of significant increase could be explained by the fact that male individuals with genes for the susceptibility for DM that are located on the X chromosome are more likely to express such a condition. It should be noticed as well that certain frequent phenotypes could have preferential advantages over others regarding adaptive value components [16]. Furthermore, different degrees of characteristic groups of traits (HRCs) between genders in the CAD group with DM led us to the possible assumption that there is a correlation between different polygene combinations that could be involved in the regulatory processes of resistance to CAD in DM patients.

So far, numerous studies have pointed out that hypertension is of multifactorial origin, including the influence of both genetic and environmental factors [21,22,23]. Despite the fact that the X chromosome is associated with lower values of BP, it is assumed that different loci for blood pressure along with several mechanisms for blood pressure regulation are present between genders [24]. In the study conducted by Hoffman et al. on rats, novel candidate genes for female-specific hypertension were proposed [24]. Furthermore, hypertension that is associated with the female gender is post-menopausal hypertension, oral contraceptive pill-induced hypertension, and pregnancy-related hypertension [25]. Our findings demonstrated that there is a significant difference in the degree of genetic homozygosity between genders in the group of CAD patients with hypertension, where significantly increased recessive homozygosity was noticed in females. The possible explanation for such findings is a decreased resistance to developmental disbalances. Furthermore, the different genetic–physiological homeostasis that exists between different genders of CAD patients with hypertension in our study could enable easier expression of hypertension as a condition in females. Also, our findings demonstrated large variations in HRC distribution between genders of CAD individuals with hypertension. These wide variations might increase the sensitivity of extreme genotype exposures to the risk of being influenced by the processes that could lead to the onset of hypertension in those individuals.

There is a limitation to this study. The combination of tested traits that we used in our study should be improved further, since the origin of genetic determination and type of inheritance ought to be better analyzed. Another limiting factor includes the number of patients, thus further study on a larger sample is advised.

In conclusion, we have demonstrated that there is an increased degree of genetic homozygosity and decreased variability for tested HRCs in CAD patients versus healthy control individuals, with different degrees of homozygosity and variability in the CAD individuals with DM versus those with HTN. Moreover, this methodology potentially might be used as a sensitive screening tool for decreased resistance to different factors that have an influence on the development of CAD. Given the facts above, our study, being among the first of its kind performed in CAD patients, provides the possibility to predict the chances to which a group of variants might be exposed in the future concerning health problems (development of the disease). This could provide an important advantage to physicians, who could then in collaboration with biochemists, physiologists, and geneticists, undertake prophylactic measures before the appearance of the symptoms. 

It would be preferable, even advisable, that HRC testing, as a type of population genetics research, is combined with next-generation sequencing tools and other biochemical traits. This approach could yield the better understanding of the origin of genetic determination as well as the nature of inheritance. Finally, our preliminary results suggest that an acceptable estimation of polygenically controlled variations could be examined in a human population.

## Figures and Tables

**Table 1:** Statistical evaluation of frequencies of homozygous recessive characteristics between groups in males and females.

Groups	Males		Females	
	z *	*p*	z *	*p*
Control/CAD	5.206	<0.001	4.220	<0.001
Control/CAD with DM	−4.331	<0.001	−2.910	0.004
Control/CAD with HTN	−4.140	<0.001	−4.199	<0.001
CAD with DM/CAD with HTN	−0.059	0.952	0.627	0.529

* Mann–Whitney *U* test.
